# Nogo receptor 1 regulates Caspr distribution at axo-glial units in the central nervous system

**DOI:** 10.1038/s41598-017-09405-9

**Published:** 2017-08-21

**Authors:** Jae Young Lee, Min Joung Kim, Lijun Li, Alexander A. Velumian, Pei Mun Aui, Michael G. Fehlings, Steven Petratos

**Affiliations:** 1 Department of Medicine, Central Clinical School, Monash University, Prahran, Victoria 3004 Australia; 2 Krembil Research Institute, Faculty of Medicine, University of Toronto, Toronto, Ontario Canada; 3 Krembil Neuroscience Centre, University Health Network, Faculty of Medicine, University of Toronto, Toronto, Ontario Canada; 4 Department of Surgery, Faculty of Medicine, University of Toronto, Toronto, Ontario Canada; 5 Department of Physiology, Faculty of Medicine, University of Toronto, Toronto, Ontario Canada; 6 Present Address: ToolGen, Inc., #1204, Byucksan Digital Valley 6-cha, Seoul, South Korea

## Abstract

Axo-glial units are highly organised microstructures propagating saltatory conduction and are disrupted during multiple sclerosis (MS). Nogo receptor 1 (NgR1) has been suggested to govern axonal damage during the progression of disease in the MS-like mouse model, experimental autoimmune encephalomyelitis (EAE). Here we have identified that adult *ngr1* ^−/−^ mice, previously used in EAE and spinal cord injury experiments, display elongated paranodes, and nodes of Ranvier. Unstructured paranodal regions in *ngr1* ^−/−^ mice are matched with more distributed expression pattern of Caspr. Compound action potentials of optic nerves and spinal cords from naïve *ngr1* ^−/−^ mice are delayed and reduced. Molecular interaction studies revealed enhanced Caspr cleavage. Our data suggest that NgR1 may regulate axo-myelin ultrastructure through Caspr-mediated adhesion, regulating the electrophysiological signature of myelinated axons of central nervous system (CNS).

## Introduction

Myelination ensures that action potentials are propagated along axons effectively. The initial contact between the central nervous system (CNS) myelin-forming oligodendrocytes and axons they ensheath, occurs at paranodal regions, which flank the node of Ranvier where voltage-gated sodium channels (Na_v_) are enriched^1^. Establishment of the node is essential so that action potentials are propagated swiftly along axons by means of saltatory conduction. Proteins enriched at paranodal regions such as contactin-1 and contactin-associated protein (Caspr), play an important role in establishing the dynamic and reciprocal relationship, from structural and functional perspectives, between the myelin extension of the mature oligodendrocyte and the axons it ensheaths, defined as the axo-glial units (for review, see refs 2, 3). Mice deficient in the gene (*cntnap1*) encoding Caspr exhibit abnormal formation of CNS nodes and this molecular architectural change may lead to reduction in conduction velocities (CVs) measured from compound action potentials (CAPs)^4^. Therefore, any ultra-structural change occurring at the paranode may influence electrophysiological properties of CNS axons.

Nogo receptor 1 (NgR1) is well-known for its inhibitory role of neurite outgrowth in the context of either CNS injury^5, 6^ or disease^7–9^. NgR1 can exert neuronal signalling upon interaction of its putative ligands such as Nogo-A, myelin-associated glycoprotein (MAG), oligodendrocyte myelin glycoprotein (OMgp) or chondroitin sulphate proteoglycan (CSPG) (for review, see ref. 10). It therefore stands to reason, that blocking NgR1-mediated signalling during CNS injury (and recently posited for disease, for review, see refs 11–14) has been regarded as a promising therapeutic strategy. However, reports suggest that NgR1 may also play a critical role in neural plasticity, highlighting that anti-NgR1 targeted therapeutics must be carefully designed to limit potential off-target effects that may include cognitive function. The dependency on functional NgR1 in synaptic activity prompted us to investigate whether this holds true for axo-glial interactions, primarily focused at the paranodal regions where fundamental axon-myelin integrity is attributable to normal saltatory conduction^15^.

We have recently shown that preservation of myelin and axons are a common finding in the spinal cord and optic nerve white matter of *ngr1* ^−/−^ mice following experimental autoimmune encephalomyelitis (EAE)-induction^9^. Although we have identified that the reduction in Nogo-A signalling in *ngr1* ^−/−^ mice is a primary cause of neuroprotection during EAE, one cannot discount that there can be ultrastructural differences between *ngr1* ^−/−^ and *ngr1* ^+/+^ mice, with possible advantages during neuroinflammation. This concept is brought into focus when we interpret recent data that illustrate potentiated neuroplasticity in *ngr1* ^−/−^ mice^15–18^ or enhanced remyelination following NgR1 antagonism in a stroke model^19^. The balance between plasticity and stability has also been identified through the generation or loss of Nogo-A-dependent strong dendritic synapses^20^. Such anatomical and physiological axo-dendritic modifications inevitably confer behavioural differences in *ngr1* ^−/−^ mice when compared to their wild type littermates^21^. However, no investigation to-date has reported similar changes in axon-myelin interactions throughout the CNS. Therefore, we have analysed the molecular organisation of axo-glial units in the CNS of the adult *ngr1* ^−/−^ mice, to ascertain whether these mice exhibit ultrastructural differences that compensate under inflammatory infiltration.

Here we report ultrastructural differences of CNS axo-glial units of adult *ngr1* ^−/−^ compared to *ngr1* ^+/+^ littermates. Firstly, both axonal diameter and myelin thickness were found to be thinner in *ngr1* ^−/−^ mice. Secondly, both paranodal and nodal lengths of *ngr1* ^−/−^ mice were elongated. Disorganisation of paranodal loops in *ngr1* ^−/−^ mice was corroborated by altered paranodal molecular domains without axonal disruption. These ultrastructural changes were attributed to alterations in the molecular organisation of the paranodal proteins that interact with the integral junctional protein, Caspr. Moreover, we found delayed and reduced CAPs in the CNS of *ngr1* ^−/−^ mice when compared with *ngr1* ^+/+^ littermate mice. The data may suggest that in *ngr1* ^−/−^ mice there exist an altered axo-glial architecture and that this may be related to Caspr distribution that is NgR1-dependent.

## Results

### Axo-glial junctions and myelin in the CNS of adult *ngr1*^−/−^ mice are unstructured in CNS major white matter tracts

**Figure 1:**
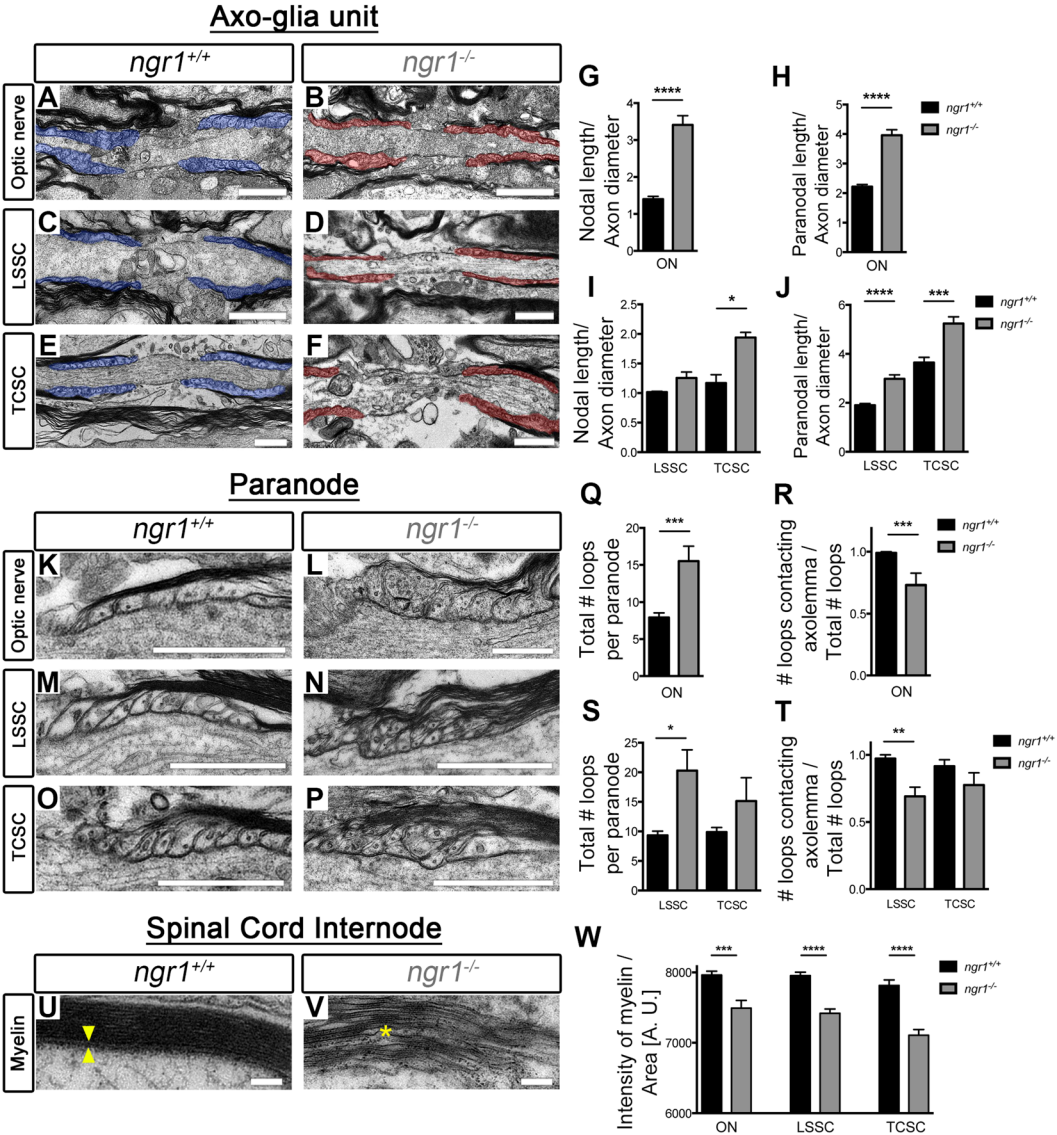
Axo-myelin ultrastructure in *ngr1* ^−/−^ optic nerves and spinal cords. (**A**–**F**) Representative EM images of longitudinal sections of (**A**,**B**) optic nerve (ON), (**C**,**D**) dorsolateral white matter LSSC, and (**E**,**F**) TCSC from *ngr1* ^+/+^ and *ngr1* ^−/−^ mice showed elongated paranodes in *ngr1* ^−/−^ (pseudo-coloured in red) compared to *ngr1* ^+/+^ (pseudo-coloured in blue) (scale bar = 1 μm). (**G**–**J**) The ratio between nodal length and axon diameter in (**G**) ON and (**I**) TCSC. (**I**–**J**) The ratio between paranodal length and axon diameter of (**I**) ON, (**J**) LSSC, and TCSC of *ngr1* ^+/+^ and *ngr1* ^−/−^ mice. (**K**–**P**) High magnification EM images of (**K**,**L**) ON, (**M**,**N**) LSSC, and (**O**,**P**) TCSC longitudinal sections showed a dysregulated formation of paranodal segments in *ngr1* ^−/−^ mice (scale bar = 1 μm). (**Q**,**S**) Total number of paranodal loops of *ngr1* ^+/+^ and *ngr1* ^−/−^ mice. (**S**,**T**) The ratio between number of paranodal loops that are in contact with the axolemma and total number of loops of *ngr1* ^+/+^ and *ngr1* ^−/−^ mice. (**U**,**V**) High magnification EM images of internodal regions of the LSSC showed a lack of intraperiodic lines (yellow arrowhead), and evident intramyelinic vacuoles (yellow asterisk) in *ngr1* ^−/−^ mice (scale bar = 100 nm). (**W**) The ratio between intensity of uranyl acetate (myelin sheath) and the area of myelin sheath (*P < 0.05, **P < 0.01 ***P < 0.001, ****P < 0.0001, *n* = 8 for both genotypes).

We specifically analysed the descending myelinated fibres of dorsolateral white matter in the thoracic-cervical (C8-T1 part of the spinal cord) and lumbosacral (L5-S1 part of the spinal cord) spinal cord (TCSC and LSSC) in naïve wild type and *ngr1* ^−/−^ mice (Fig. 1). We prepared longitudinal ultra-thin sections (100 nm) from the dorsolateral white matter in LSSC and TCSC, as well as the optic nerve, (a CNS white matter area where neuroinflammation occurs readily during EAE and MS), for transmission electron microscopy (TEM) (Fig. 1). The ultrastructure of nodal and paranodal regions (pseudo-coloured to blue in *ngr1* ^+/+^ and to red in *ngr1* ^−/−^) was examined and the ratios of paranodal and nodal widths along with axon diameters were quantified (Fig. 1A–J). As axonal diameter has been shown to regulate the structure of axo-glial units, including the paranodal length^22^, we examined the ratio of paranodal length to axon diameter, and found that it was increased in all three CNS white matter regions of *ngr1* ^−/−^ compared to *ngr1* ^+/+^ mice (Fig. 1H and J). Furthermore, the ratios generated between nodal lengths and axon diameters were increased in optic nerves and TCSC (an increased trend was also observed for the LSSC, though not statistically significant; Fig. 1G and I).

An in-depth analysis of the paranodal regions revealed a ‘disorganised layering’ pattern of paranodal loops in the CNS sections from *ngr1* ^−/−^ compared to *ngr1* ^+/+^ mice (Fig. 1K–T). We counted the total number of loops, and the number of paranodal loops not in contact with the axolemma and found that the total number of paranodal loops per one paranode, along with the fraction of the loops contacting the axolemma were considerably higher in *ngr1* ^−/−^ when compared with *ngr1* ^+/+^ mice (Fig. 1Q–T). Moreover, obvious gaps between the intraperiodic lines of myelin sheaths in the spinal cords of *ngr1* ^−/−^ compared to tightly compacted myelin sheaths were observed in the *ngr1* ^+/+^ mice (Fig. 1U and V). Quantification of the compactness of myelination through measurement of the intensity of uranyl acetate staining showed intramyelinic vacuolisation within the myelin sheaths of descending spinal cord white matter fibres from *ngr1* ^−/−^ mice (Fig. 1W). These data indicate that myelination is incomplete in the CNS of *ngr1* ^−/−^ mice due to inappropriate formation of the paranode and a lack of compaction.

Axonal neurofilament spacing has been shown to have an impact on axon diameter^23^. Therefore, we measured distance between neurofilaments of axons from LSSC of the *ngr1* ^+/+^ and *ngr1* ^−/−^ mice, however, we found no differences of neurofilament spacing between *ngr1* ^+/+^ and *ngr1* ^−/−^ mice (Fig. S1). These results indicate that NgR1 controls axon diameter developmentally without altering neurofilament spacing. Although abnormal paranodal ultra-structure may be evident in the CNS of *ngr1* ^−/−^ mice, the axons were intact and we could not detect any differences in the organisation of neurofilaments nor distance between neighbouring neurofilaments of myelinated axons of spinal cords (Fig. S1)^24^.

### Thinner axons and myelin sheaths in the CNS of *ngr1*^−/−^ mice

**Figure 2:**
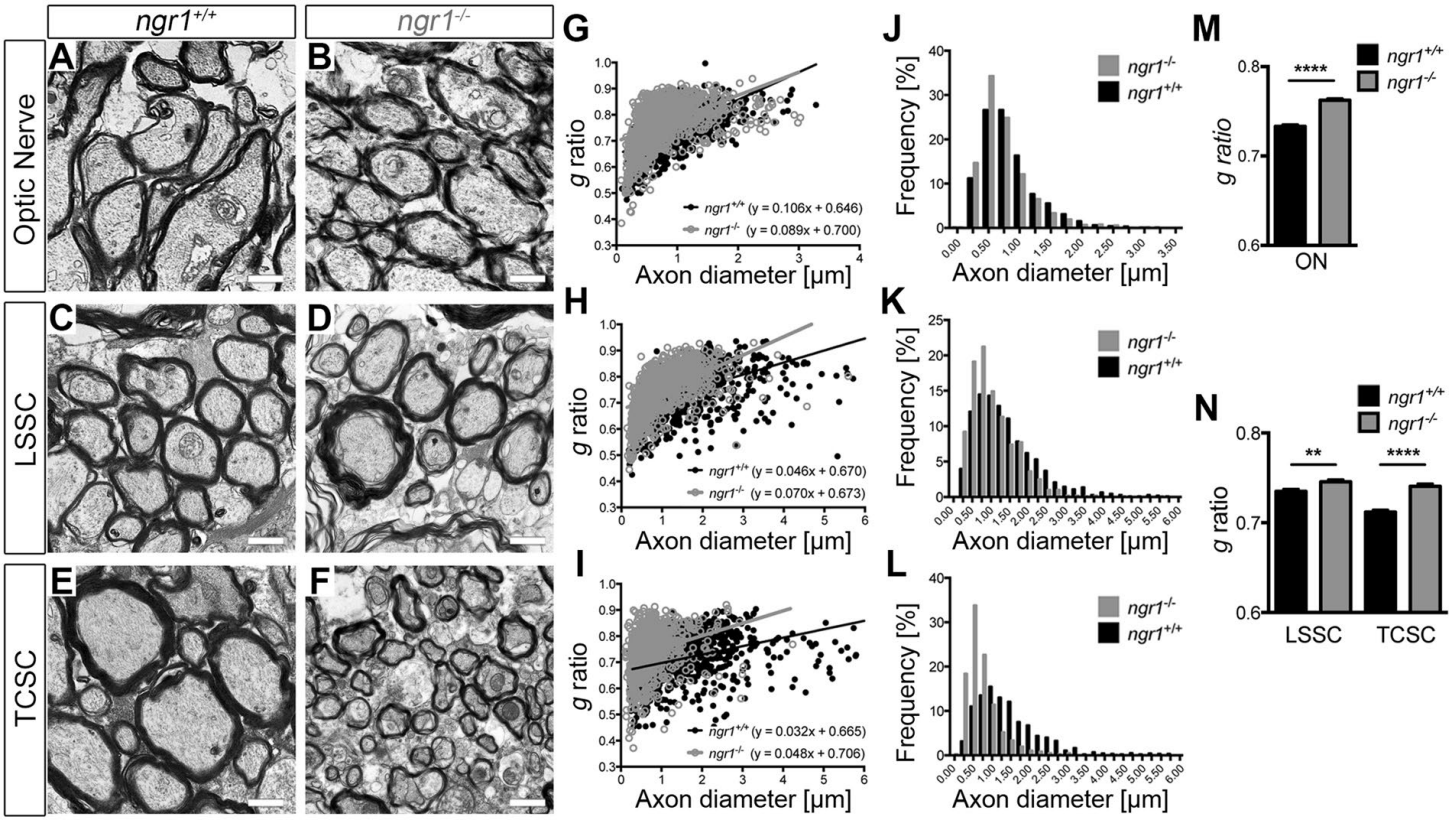
Thinner axon diameter and myelin thickness in *ngr1* ^−/−^ mice. (**A**–**F**) EM images of ON (**A**,**B**), dorsolateral white matter of LSSC (**C**,**D**), and TCSC (**E**,**F**) cross sections of *ngr1* ^+/+^ and *ngr1* ^−/−^ mice (scale bar = 1 μm). (**G**–**I**) Scatter plots display *g* ratios of individual myelinated axons as a function of the respective axon size and the linear regression of the *g* ratio measurements for each genotype is shown for the three CNS areas. (**J**–**L**) The percentage of myelinated axons with respect to axon diameter at 0.25 μm intervals for the three CNS areas. (**M**,**N**) The mean *g* ratios of *ngr1* ^+/+^ and *ngr1* ^−/−^ mice (**P < 0.01, ****P < 0.0001, *n* = 8 for both genotypes).

Since we identified that there exist substantial differences within the organisation of myelin in the *ngr1* ^−/−^ CNS with regards to paranodal junctions and myelin compaction, we hypothesised that the thickness of the myelin sheaths may also be reduced. To examine this directly, we prepared transverse sections of dorsolateral white matter regions of TCSC, LSSC and optic nerve for TEM analysis (Fig. 2). We measured the axon diameter and fibre diameter (axon with myelin) to determine the *g* ratio, and found that it was substantially elevated in *ngr1* ^−/−^ compared to *ngr1* ^+/+^ spinal cords and optic nerves (Fig. 2). While this resulted from thinner myelin relative to the axon diameter, we also observed a larger number of small diameter axons in *ngr1* ^−/−^ compared to *ngr1* ^+/+^ mice (Fig. 2J–L). As the axon diameter is intimately linked to myelin thickness^25^, our findings suggest that reduced axon diameter in the CNS of *ngr1* ^−/−^ mice may be related to the incomplete myelination.

### Altered myelin ultrastructure is associated with increased Caspr distribution in the CNS of *ngr1*^−/−^ mice

**Figure 3:**
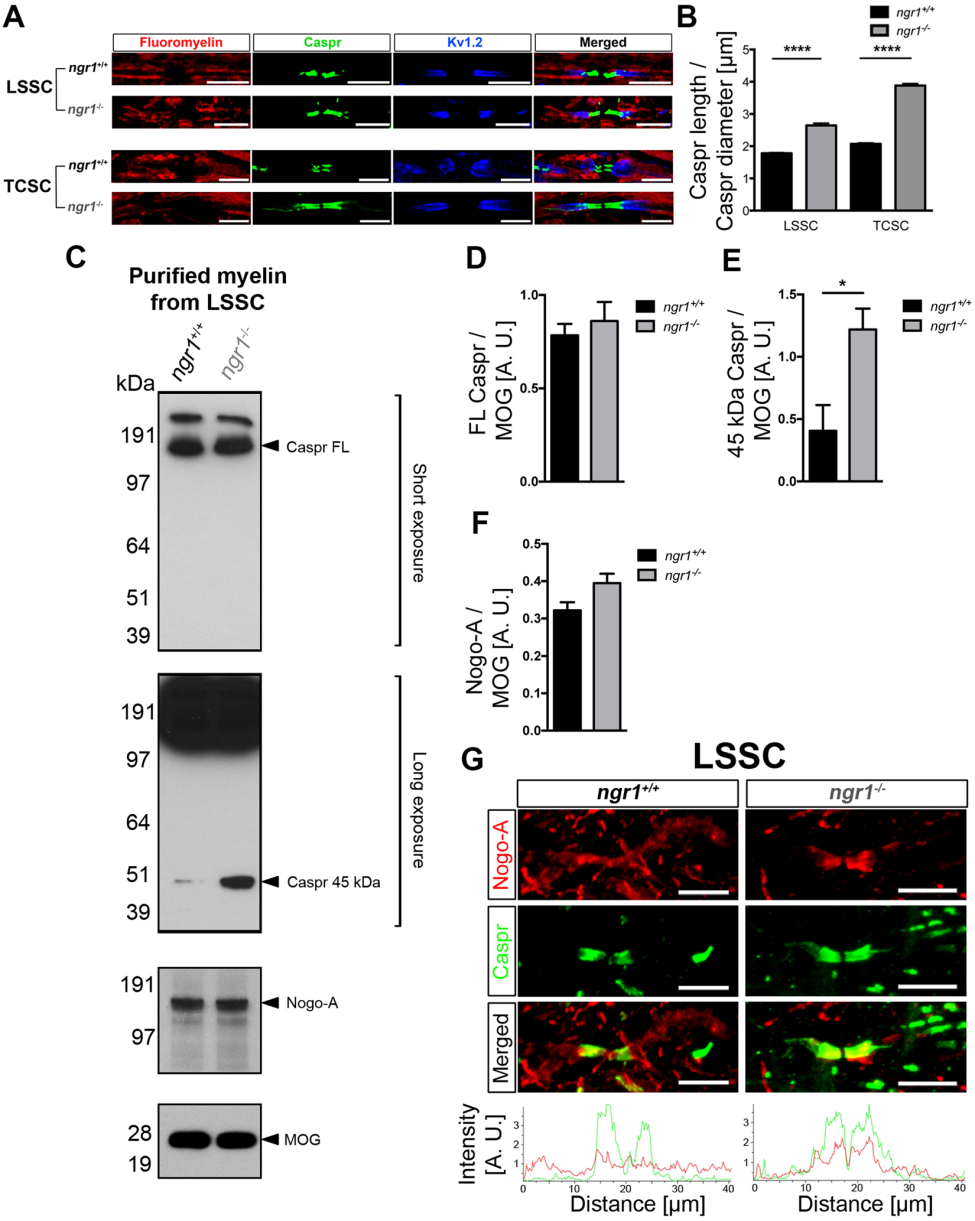
Increased levels of cleaved form of Caspr in *ngr1* ^−/−^ spinal cord lysates. (**A**,**B**) Immunofluorescent images of dorsolateral white matter of LSSCs showed longer Caspr + paranodal length in *ngr1* ^−/−^ mice. (**A**) Myelin was identified with fluoromyelin, paranodes were identified with Caspr, and juxtaparanodes were identified with K_v_1.2 (scale bar = 10 μm). (**B**) The ratio between Caspr length and diameter (****P < 0.0001, *n* = 4 for both genotypes). (**C**–**F**) Western blot of purified myelin of LSSCs from *ngr1* ^+/+^ and *ngr1* ^−/−^ mice showing an increased cleaved form of Caspr (~45 kDa) in *ngr1* ^−/−^ and unaltered Nogo-A levels between *ngr1* ^+/+^ and *ngr1* ^−/−^ (MOG served as a loading control). (**C**) Representative immunoblots for Caspr, Nogo-A, and MOG of purified myelin. (**D–** **F**) Densitometric quantification of (**D**) full-length Caspr (FL-Caspr); (**E**) 45 kDa degradation product of Caspr; (**F**) Nogo-A and MOG (Mean ± SEM, *P < 0.05, *n* = 3 for both genotypes). (**G**) Immunofluorescent images of dorsolateral white matter of LSSCs of *ngr1* ^+/+^ and *ngr1* ^−/−^ mice showed different Nogo-A localisation at the axo-glial junction. The intensity profile of immunostaining showed distinct co-localisation of paranodal Caspr and Nogo-A in *ngr1* ^−/−^ whereas punctate but constant Nogo-A immunostaining throughout the axo-glial units and internodes in *ngr1* ^+/+^ (scale bar = 10 μm).

The ultrastructural changes in the axo-glial units within the CNS of *ngr1* ^−/−^ mice prompted our investigation for potential modifications to integral paranodal adhesion molecules. We performed immunolabelling analyses for Caspr (to stain the paranode), voltage-gated potassium channel type 1.2 (K_v_1.2) (to stain the juxtaparanode) and fluoromyelin (to stain the internode). In keeping with the TEM analysis, we demonstrated an increased length of the Caspr-positive paranodes measured in both the LSSC and TCSC in *ngr1* ^−/−^ when compared to *ngr1* ^+/+^ littermate mice (Fig. 3A and B). Although there is a substantial lengthening of paranodal Caspr, unlike paranodal mutant models such as the *cntnap1* mutant mice^4, 26^, we could not detect an altered distribution of the juxtaparanodal K_v_1.2 or the sodium channel using pan-Na_v_ staining (Fig. 3A,C and D).

Since ultrastructural difference exists in the CNS myelin of *ngr1* ^−/−^ mice that correlate with decreases in the composition of integral axo-glial proteins, we hypothesised that the defect in the *ngr1* allele, would act to modify the metabolically active myelin membrane. For this, we prepared myelin fractions of naïve LSSCs from both *ngr1* ^+/+^ and *ngr1* ^−/−^ mice, then performed western blot analysis to identify myelin and axo-glial proteins in their bound or modified state. Although the expression level of full-length Caspr did not differ between *ngr1* ^+/+^ and *ngr1* ^−/−^ CNS myelin, a cleaved product of Caspr was increased in *ngr1* ^−/−^ compared with *ngr1* ^+/+^ mice, which may account for the distributed Caspr expression (elongated Caspr-positive domains) in the CNS of *ngr1* ^−/−^ mice (Fig. 3E–H).

**Figure 4:**
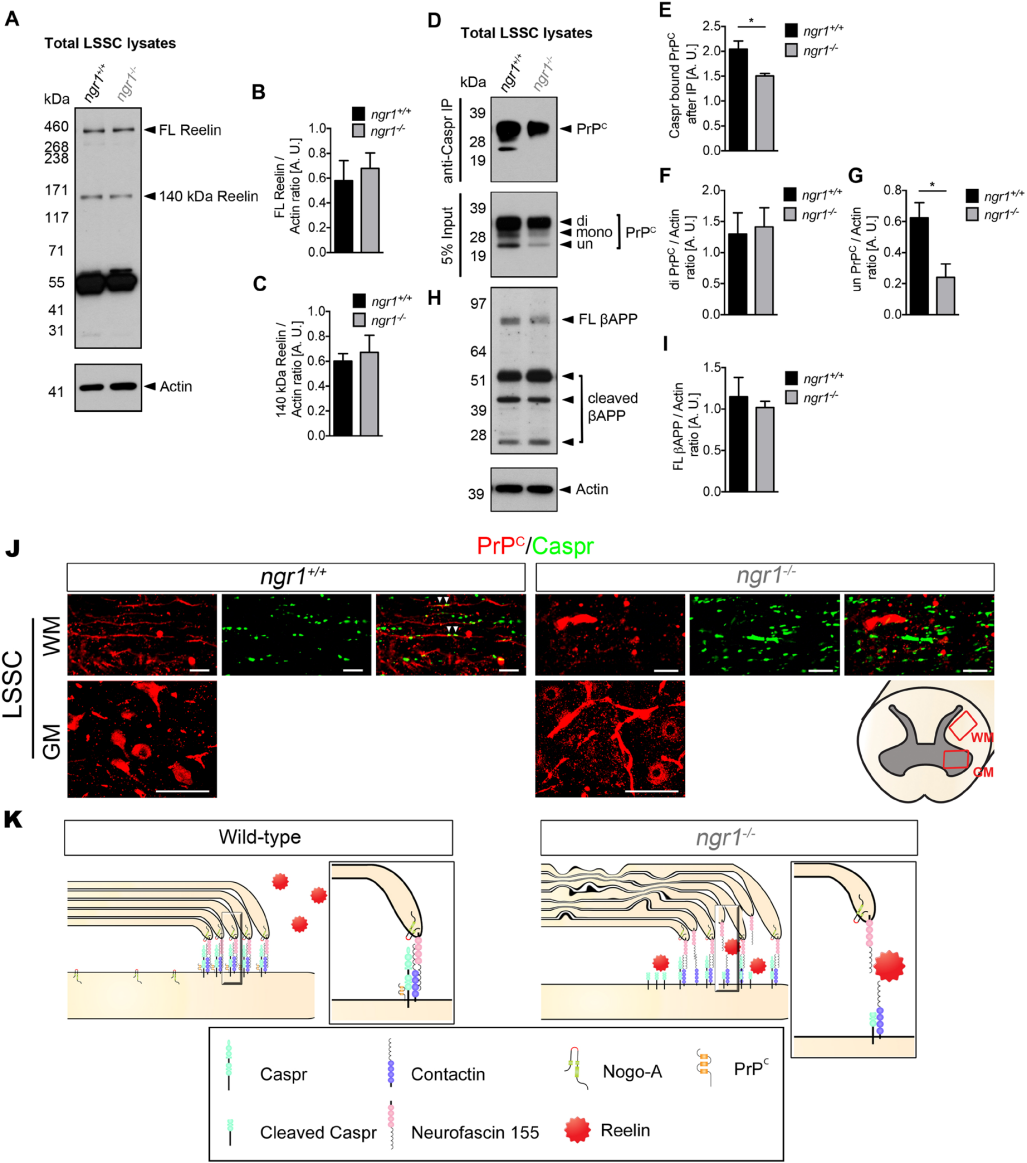
Reduced interaction between PrP^C^ and Caspr in *ngr1* ^−/−^ mice. (**A**–**C**) Western blot of LSSC lysates from *ngr1* ^+/+^ and *ngr1* ^−/−^ mice showed no difference in Reelin expression. (**A**) Representative immunoblots for Reelin and Actin loading control. (**B**) Densitometric quantification of full-length Reelin (FL Reelin) and (**C**) 140 kDa degradation product of Reelin and Actin respectively. (**D**) Immunoprecipitation of Caspr showed reduced interaction with PrP^C^ in LSSC from *ngr1* ^−/−^ mice. Western blot for PrP^C^ from 5% input of pre-immunoprecipitation sample showed three bands which represents the three glycosylation states of PrP^C^; di-glycosylated (di), mono-glycosylated (mono), and un-glycosylated form (un), respectively). (**E**) Densitometric quantification of total PrP^C^ and Caspr bound PrP^C^. (**F**) Densitometric quantification of di-glycosylated PrP^C^, (**G**) unglycosylated PrP^C^. (**H**) Representative immunoblots for βAPP, and Actin loading control. (**I**) Densitometric quantification of βAPP and Actin (*P < 0.05, *n* = 4 for both genotypes). (**J**) Immunofluorescent images showed axonal PrP^C^ and co-localisation with Caspr within LSSC white matter (WM) of *ngr1* ^+/+^ whereas, lack of axonal PrP^C^ staining in WM was found in *ngr1* ^−/−^ mice. PrP^C^ expression in motor neuronal soma within the gray matter (GM) of LSSC was found in both genotypes. Illustration showing where the WM and GM images were taken from LSSCs are shown at the right-hand side (WM scale bars = 10 μm; GM scale bars = 50 μm). (**K**) In wild-type, paranodal junction is tightly organised by the putative interaction among glial Neurofascin 155, axonal Caspr and Contactin. Furthermore, tight interaction between Caspr and PrP^C^ inhibits Reelin-mediated Caspr cleavage, which allows for tight compaction of paranodal loops and myelin sheath. In the absence of NgR1, reduced interaction between PrP^C^ and Caspr mediates Reelin-mediated cleavage of Caspr, which leads to de-compaction of paranodal loops and myelin sheath. Furthermore, paranodal localisation of Nogo-A in *ngr1* ^−/−^ whereas broad expression of Nogo-A throughout the axo-glial unit is found in wild-type.

Caspr is expressed in the mammalian CNS in its native and cleaved form^27^. So far, γ-secretase^28^ and Reelin^29^ have been shown to be associated with the cleavage of Caspr. Therefore, we analysed the activity of these enzymes by identifying their substrates and products within the CNS of naïve *ngr1* ^+/+^ and *ngr1* ^−/−^ mice. First, we analysed the expression level of β-amyloid precursor protein (βAPP), since γ-secretase is well-known for its role in amyloid-β (Aβ) generation through the cleavage of βAPP^30^. We demonstrated that the levels of full-length βAPP were not different between the naïve LSSC of *ngr1* ^+/+^ and *ngr1* ^−/−^ mice, possibly excluding the involvement of γ-secretase in the observed increase of Caspr cleavage occurring in the *ngr1* ^−/−^ spinal cord (Fig. 4H and I). It has been suggested that Reelin can act as a serine-protease, cleaving fibronectin or laminin at several sites^31^. As Caspr contains four laminin-G-like domains, Reelin-mediated cleavage of Caspr has been suggested. Indeed, Reelin was shown to cleave Caspr thereby generating the 45–50 kDa truncated product at the laminin-G-like domains with an intermediate degradation product at ~100 kDa^29^. Moreover, it was recently demonstrated that the cellular prion protein, PrP^C^ can interact with Caspr to inhibit this cleavage event^29^. Therefore, we investigated the expression of these molecules localised to myelin and axons and found that the interaction between Caspr and PrP^C^ is considerably reduced in *ngr1* ^−/−^ compared with *ngr1* ^+/+^ LSSC, although no differences were observed in the levels of full-length or the active proteolytic form of Reelin (140 kDa)^31^ (Fig. 4A–G), suggesting a possible increase in Caspr shedding within the CNS of *ngr1* ^−/−^ mice. Furthermore, there was a substantial reduction in the unglycosylated form of PrP^C^ in the *ngr1* ^−/−^ LSSC. Since the unglycosylated form of PrP^C^ has been reported to be enriched in myelin and myelinic PrP^C^ may participate in the maintenance of myelin against PrP-associated pathology^32, 33^, we posited that the reduction in the unglycosylated form of PrP^C^ may indeed have resulted from reduced myelin PrP^C^ expression in the CNS of *ngr1* ^−/−^ mice. However, we did not detect a difference in the level of PrP^C^ expression in purified myelin of *ngr1* ^−/−^ compared to *ngr1* ^+/+^ spinal cords, ruling out this possibility (Fig. S2A, C and D). To further address the difference in PrP^C^ expression within spinal cords, we performed PrP^C^ immunostaining in longitudinal sections demonstrating descending fibre tracts (dorsal white matter; WM) and motor neuronal somata (gray matter; GM). Strikingly, PrP^C^ immunostaining showed a near absence of WM axonal PrP^C^ in spinal cords of *ngr1* ^−/−^, whereas prominent axonal PrP^C^ was abundantly found within WM of *ngr1* ^+/+^ spinal cords as well as its co-localisation with Caspr (Fig. 4J). On the other hand, we found no differences in PrP^C^ expression in motor neuronal somata in the GM of spinal cords between *ngr1* ^+/+^ and *ngr1* ^−/−^ mice, suggesting PrP^C^ protein is produced in neurons, however its localisation to white matter axons seems impaired in *ngr1* ^−/−^ mice. Furthermore, as Reelin is reported to be expressed on oligodendrocytes, we addressed the possibility of differences in Reelin levels in purified CNS myelin from *ngr1* ^−/−^ to *ngr1* ^+/+^ mice. We could not detect the 140 kDa proteolytic form of Reelin in purified myelin and there was no difference in full-length Reelin in *ngr1* ^−/−^ compared to *ngr1* ^+/+^ mice (Fig. S2B and E). Therefore, the inference may be that the significant reduction of PrP^C^ observed in *ngr1* ^−/−^ CNS is axonal due to the impairment of its transport from the neuronal soma to its axon and may implicate this as a mechanism of unabated Reelin activity.

Despite the elevated levels of cleaved Caspr observed in the spinal cords of *ngr1* ^−/−^ mice, we did not find differences in other paranodal proteins, such as neurofascin 155 (Fig. S3B and C). These data may indicate that the disrupted paranodal domain formation in *ngr1* ^−/−^ CNS axo-glial units maybe a result of increased Caspr cleavage. Despite this, we did not find differences in juxtaparanodal proteins, such as K_v_1.2 (Fig. S3B and D). Moreover, we did not detect any differences in the levels of myelin proteins such as myelin basic protein (MBP), 2′, 3′-Cyclic-nucleotide 3′-phosphodiesterase (CNPase), myelin oligodendrocyte-glycoprotein (MOG), native nMAG (Fig. S3B and E–G) or indeed Nogo-A in myelin prepared from LSSCs of *ngr1* ^−/−^ compared with *ngr1* ^+/+^ mice (Fig. 3E and H). However, the expression pattern of Nogo-A was found to be different between *ngr1* ^+/+^ and *ngr1* ^−/−^ mice LSSCs. We showed that Nogo-A was localised at the CNS paranodes in *ngr1* ^−/−^ mice, whereas it was distributed throughout the entire axonal and internodal segments including the paranodes in *ngr1* ^+/+^ spinal cord WM (Fig. 3I), which suggests that NgR1 may regulate the localisation of Nogo-A expression as observed for PrP^C^ within the axo-glial units.

To summarise our proteophenotypic findings, we propose that the absence of NgR1 may result in impairment of axonal localisation of PrP^C^ from the neuronal soma, which enhances Reelin-mediated cleavage of Caspr, thereby resulting in unstable paranodal loops and incomplete myelin formation. Lacking NgR1 is also likely to modulate the expression pattern of Nogo-A along the internodes (Fig. 4K).

### Electrophysiological evidence for slowed axonal conduction velocity in the CNS of *ngr1*^−/−^ mice

**Figure 5:**
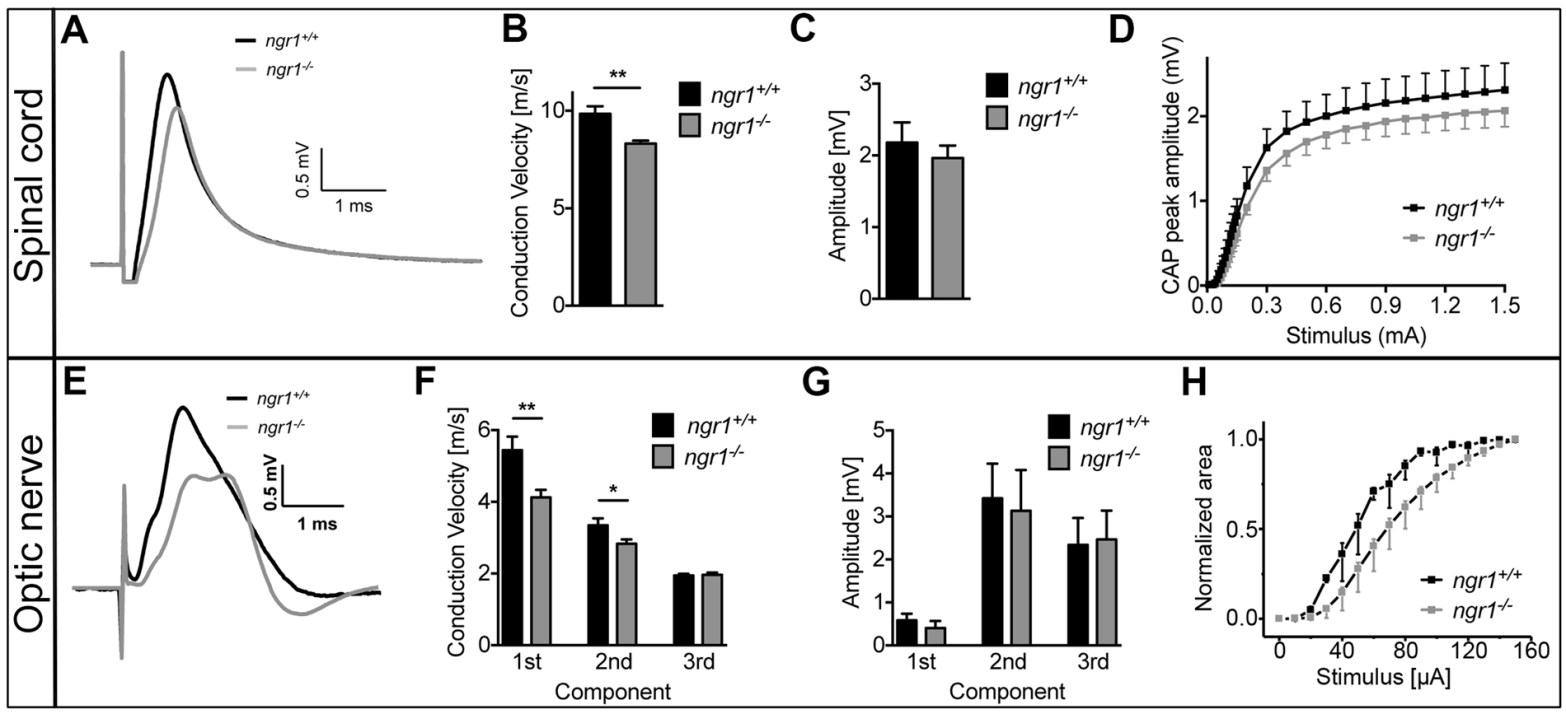
Delayed latencies of CAP recordings from the CNS of *ngr1* ^−/−^ mice. (**A**) Representative recordings of CAP series from naïve spinal cords from *ngr1* ^+/+^ and *ngr1* ^−/−^ mice as measured by sucrose gap showed delayed CV in *ngr1* ^−/−^. (**B**) CVs and (**C**) amplitudes were measured at peak maxima. (**D**) Axon recruitment profiles for the peak amplitude. (**F**) Representative recordings of CAP series from naïve optic nerves from *ngr1* ^+/+^ and *ngr1* ^−/−^ mice as measured by suction electrodes showed delayed CV in *ngr1* ^−/−^. (**F**) CVs and (**G**) amplitudes were measured at peak maxima. (**H**) Axon recruitment profiles for the peak amplitude (*n* = 6 for *ngr1* ^+/+^ and *n* = 9 for *ngr1* ^−/−^).

Given the substantial differences observed in the ultrastructure and biochemistry of *ngr1* ^−/−^ myelin and in particular, the axo-glial unit, we interrogated the electrophysiological profiles of naïve *ngr1* ^−/−^ mice and compared these with *ngr1* ^+/+^, by recording CAPs from spinal cord WM and the optic nerves using a modified single sucrose gap chamber for spinal cords^34^ and suction electrodes for the optic nerves. We measured the CAP peak amplitudes and area that may be attributable to changes in numbers of conducting axons and their calibres^35, 36^, then calculated the CVs from peak latencies that may be attributable to changes in axon calibres and myelination^37, 38^ as well as to paranodal specialisations^39, 40^. We were able to demonstrate after measurement that a significant reduction in CV occurs in the spinal cords of *ngr1* ^−/−^ compared to *ngr1* ^+/+^ mice (Fig. 5A and B). However, the maximum amplitude of CAPs did not differ significantly between *ngr1* ^−/−^ and *ngr1* ^+/+^ spinal cord WM axons (Fig. 5A, C and D), which suggests that there is no difference in the number of CNS myelinated axons. To confirm this, we applied 4-aminopyridine (4-AP, a K_v_ blocker which has minor effects on myelinated axons in normal conditions and preferentially blocks the K_v_ in unmyelinated or injured/demyelinated axons)^41^ before recording CAPs. We found no significant differences in the 4-AP effects on CAPs from the spinal cord WM between *ngr1* ^−/−^ and *ngr1* ^+/+^ mice (Fig. S5A–C).

Since optic nerve axons in mice are all myelinated, CAP recordings of these nerves are extensively used for characterising the function of CNS myelinated axons. Optic nerve CAPs, recorded with suction electrodes, exhibited three distinct peaks, which is in line with previously published results^42, 43^ (Figs 5E and S5). These three components are thought to represent fast-conducting large diameter, intermediate-conducting medium, and slow-conducting small diameter myelinated axons^44, 45^. Optic nerve CAPs are not always uniform in terms of their three peaks, and in fact only 9/14 optic nerves in our recordings from *ngr1* ^+/+^ mice exhibited three distinct CAPs (Figs 5E and S5D–F). Nevertheless, comparing the *ngr1* ^−/−^ optic nerve CAPs with those in *ngr1* ^+/+^ showed that the latencies of the first and second components are significantly delayed in *ngr1* ^−/−^ mice, with accordingly slower CVs (Figs 5E,F and S5G–L). However, the CVs from the third CAP components that represent the smallest myelinated axons, did not differ between *ngr1* ^−/−^ and *ngr1* ^+/+^ optic nerves (Fig. 5E and F). These results indicate that there exists a slower conduction in medium and large calibre retinal ganglion cell axons in *ngr1* ^−/−^ mice. We did not detect any significant change in the peak amplitudes of CAP components between *ngr1* ^−/−^ and *ngr1* ^+/+^ mice, which corroborates data obtained from the spinal cord recordings (Figs 5E, G, H and S5G and J), given the larger axon calibres in the spinal cord (1–2 μm)^46^ compared to optic nerves where the diameters of nearly 80% of the axons are <0.75 μm^43^.

## Discussion

Here we show that NgR1 plays an active role in regulating and maintaining the axo-myelin unit within the CNS of adult mice that is commonly disrupted during EAE and MS^47^. Moreover, we have identified that *ngr1* ^−/−^ mice exhibit incomplete formation of axo-glial units without disrupting the distribution of ion channels and, in essence, preserving axonal integrity, a phenomenon observed for other cell adhesion knockout phenotypes such as *cadm4* ^−/−^^48^ and to a lesser extent *mag* ^−/−^ mice^49^. This morphological outcome identified in the CNS of *ngr1* ^−/−^ mice may be governed through a reduced Caspr-PrP^C^ interaction, possibly leaving Reelin unopposed to cleave Caspr^29^. Despite the enhanced cleavage of Caspr, the full-length isoform remains strongly expressed arguing for a continuous turnover of the integral paranodal protein, possibly providing further insights into the neuroplasticity-dependent role of NgR1^15^. This physiological role of NgR1 at the axo-myelin junctions may be of substantial importance during MS progression since denuded axons potentially through the loss of paranodal integrity governs the heightened expression of NgR1, driving axonal damage.

Under physiological conditions, compact myelin is fundamental for the fast propagation of electrical signals. The dynamic paranodal ultrastructure of the axo-myelinic unit, segregates the Na_v_ at the nodes of Ranvier from the clustered K_v_ at the juxtaparanode. However, in animal models of various inherited and acquired disorders, disruption in the integrity of myelin may slow the axonal conduction or can result in conduction block amongst affected fascicles (for review see ref. 47). Moreover, in chronic hypomyelinated or demyelinated states the distribution of these ion channels along the denuded axolemma may potentiate axonal damage^50, 51^. To provide the compartmentalisation of clustered ion channels, paranodal myelin consists of several key adhesion proteins that establish and maintain these electrophysiologically-active domains. Chief among these is Caspr, whose expression we found to be maintained within the CNS of *ngr1* ^−/−^ mice along with preserved axonal integrity. These data may suggest that NgR1 plays an active role in axo-glial dynamics, previously attributed to regenerating growth cones^5^.

Mechanistic insight in the importance of Caspr for axonal integrity has been derived from the eloquent description of its importance in the transverse bands comprising of the Caspr/contactin complex that regulates the width of the paranodal axo-glial junction gap. *cntnap1*-null mice^4^, or indeed *cntn1*-nulls^52^, display severe neurological deficits with profound axonal dystrophy throughout the CNS. However, in the *shaking* or *shiverer* (*mbp* mutant) models of dysmyelination, the transverse bands are to a large degree intact^53, 54^, with reduced neurological severity and axonopathy. Intriguingly, these myelin mutants display Na_v_ segregation from K_v_^51, 55^ that resemble the lack of lateral redistribution that is observed in our *ngr1* ^−/−^ mice. Moreover, the recently reported human homozygous frame-shift mutation of *cntnap1* has identified significant reduction in CV congruent with elongated nodes of Ranvier and hypomyelinated nerve fibres^56^, highlighting the importance of Caspr in maintaining myelinated axonal function. However, during CNS inflammation, *ngr1* ^−/−^ mice maintain axonal levels of full-length Caspr possibly due to the developmentally imposed continued expression as a consequence of unabated Reelin activity. A plausible hypothesis for this physiological compensatory mechanism of Caspr in axons may be the continued turnover of myelin in the *ngr1* ^−/−^ mice, although this would require further determination.

Despite this, it has been previously established that Reelin-mediated shedding from the neuronal plasma membrane is inhibited by PrP^c^ thereby modulating neurite outgrowth potential^29^, a physiological attribute of NgR1^5^. The mechanism by which Caspr regulates neurite outgrowth inhibition is somewhat unclear although it was touted that PrP^c^ can directly bind Caspr limiting its shedding from the neuronal membrane by the serine protease activity of Reelin^29^. This molecular mechanism of neurite outgrowth inhibition was ratified following the observations that *cntnap1* ^−/−^ cerebellar neurons were longer than wild type neurons and this effect was emulated in *prnp* ^−/−^ (PrP^C^) neurons. Of course, one potential caveat to this observation is that these are isolated cerebellar neurons whose axons are not myelinated and so physiological axo-myelinic units have not been established in these assays. However, these investigators did go on to show that increased Caspr proteolysis (in *prnp* ^−/−^ mice) correlates with increased locomotor recovery following spinal cord injury, again a compensatory response germane to *ngr1* ^−/−^ mice following either injury or inflammatory challenge^9, 57^.

Regardless of the regenerative role documented for *prnp* ^−/−^ mice following spinal cord injury, it was recently reported that EAE-induction of these mice may exacerbate the disease with a more severe axonopathy exhibited^58^. Although at first counterintuitive to the argument of limited Caspr shedding from the neuronal plasma membrane maintaining axonal integrity under neuroinflammatory conditions, our finding that deletion of the *ngr1* allele preserved axons can be explained by the continued presence of PrP^c^ in CNS neurons but altered localisation and distribution of PrP^c^. Indeed, neuronal PrP^c^ was observed more closely localised to the neuronal somata than dispersed through to the axolemma and axo-myelinic junctions in the *ngr1* ^−/−^ when compared with *ngr1* ^+/+^ mice.

Our current findings have defined a novel role for NgR1, by virtue of an indirect regulation for axo-glial unit formation, established through the modulation of axonal expression of PrP^C^ and leading to an unabated Reelin-mediated Caspr cleavage. Reelin-mediated Caspr cleavage may in turn result in continual turnover of myelin supported by our observation of thinner myelin within the CNS of *ngr1* ^−/−^ mice. To ascertain the role of NgR1 in myelin turnover and potentially remyelination, experiments inducing demyelination without axonal pathology such as lysolecithin-induced demyelination may be required. Indeed, as a recent report suggested for an active role of NgR1-signalling in myelin repair during white matter stroke, abrogating NgR1-signlaing may be a viable option for regenerative medicine in other CNS inflammatory diseases such as MS.

## Methods

### Animals

All experiments were conducted according to the Alfred medical research and education precinct (AMREP), Monash University and University of Toronto guidelines for animal experiments. *ngr1* ^−/−^^59^ and *ngr1* ^+/+^ mice on a C57Bl/6 background were bred and maintained at the AMREP animal facility. All animal experiment protocols were performed in accordance with and approved by the Monash University Animal Ethics Committee (AEC# MARP/2011/128 and AEC #E/1532/2015/M) and University Health Network Animal Care Committee (AUP# 2578). All animal experiments were in accordance with the National Health and Medical Research Committee (NHMRC) of Australia guidelines and regulations that is governed by the Australian Code for the care and use of animals for scientific purposes 2013 and complies with the Victorian Cruelty to Animals Act 1986. For experiments, age-matched (P120-140) and sex-matched (female) *ngr1* ^+/+^ and *ngr1* ^−/−^ mice were utilised.

### Electron microscopy

*ngr1* ^+/+^ and *ngr1* ^−/−^ mice were deeply anesthetised with CO_2_ and perfused with PBS followed by 2.5% glutaraldehyde and 4% paraformaldehyde. The optic nerves, lumbar and sacral (lumbosacral) and thoracic and cervical spinal cord were dissected and post-fixed in 2.5% glutaraldehyde and 4% paraformaldehyde for 24 hours at 4 °C. Tissues were then washed in 0.1 M cacodylate buffer and contrasted with 1% osmium tetroxide in 0.2 M cacodylate buffer for 2 hours at room temperature. Tissues were then washed in 0.1 M cacodylate buffer and dehydrated in a sequential ethanol gradient from 50–100% and embedded longitudinally or transversely in Epon. Prior to sectioning, all embedded tissue was coded and stored by a different investigator listed in the authorship and the senior investigator. The subsequent analysis was thereby performed blinded to exclude bias. Semi-thin sections (0.9 μm) were then prepared and stained with toluidine-blue and analysed with an Olympus dotslide BX51 microscope with 20x objective lens (Olympus). From toluidine-blue stained longitudinal sections of both lumbosacral and thoracic-cervical spinal cord, internodal lengths and axonal diameters were measured (at least 500 axons were measured from each mouse, *n* = 8 for both *ngr1* ^+/+^ and *ngr1* ^−/−^). Ultra-thin sections (100 nm) were cut and stained with 1% aqueous uranyl acetate. Stained sections were analysed either under 20,000x magnification on a transmission electron microscope (Hitachi H-7500) for *g* ratio calculation or 50,000x magnification for paranodal ultrastructure, and all images were captured with a Gatan digital camera (model 791). Axonal diameters (between the range of 0.25 to 6 μm) and the *g* ratio from transverse sections were determined as previously described^60^. At least 300 axons were measured from each section. Paranodal and nodal ratios were measured by calculating the ratio of paranodal or nodal length over axonal diameter from longitudinal sections (at least 100 paranodes/nodes were measured from each mouse; *n* = 8 for both *ngr1* ^+/+^ and *ngr1* ^−/−^). Distance between neighbouring neurofilaments were measured from high magnification images from lumbosacral spinal cords (at least 100 axons were measured from each mouse; n = 8 for both *ngr1* ^+/+^ and *ngr1* ^−/−^). All measurements were performed manually using ImageJ (Fiji) software.

### Immunohistochemistry

*ngr1* ^+/+^ and *ngr1* ^−/−^ mice were deeply anesthetised with CO_2_ and perfused with PBS followed by 4% paraformaldehyde. The spinal cords were dissected and post-fixed in 4% paraformaldehyde (in 0.1 M phosphate buffer) for 24 hours at 4 °C. Samples were then cryoprotected in 30% sucrose for 24 hours at 4 °C. Samples were then embedded in O.C.T. medium (Tissue-Tek) and frozen in isopentane cooled on dry ice. Longitudinal sections were cut at 10 μm on a cryostat (Leica Microsystems) and mounted on microscope slides. Sections were then post-fixed for 7 minutes with cold methanol (−20 °C) and washed with PBS. Sections were then blocked and permeabilised in 10% normal goat serum and 0.3% Triton X-100 in PBS for 1 hour at room temperature. Primary antibodies diluted in blocking buffer (5% normal goat serum and 0.1% Triton X-100) were applied on tissue sections for 24 hours at 4 °C. Secondary antibodies diluted in blocking buffer were applied on tissue sections for 1 hour at room temperature. Sections were counterstained with Fluoromyelin Red (Invitrogen) for 1 hour at room temperature, and then mounted with fluorescent mounting medium (Dako). Images were taken using a Nikon A1 Eclipse confocal microscope with ×60 oil immersion objective (NA = 1.4). The captured multichannel images were separated into individual channels by ImageJ (Fiji) and assembled and formatted using Adobe Photoshop.

Following primary antibodies were used: Rabbit anti-Caspr (Cat. ab34151, Abcam, 1:1,000); Mouse anti-Caspr (Cat. ab105571, abcam, 1:1,000); Mouse anti-Kv1.2 (Cat. ab98970, Abcam, 1:1,000); Rabbit anti-Nogo-A (Cat. Ab5888, Millipore, 1:200); Mouse anti-PrP^C^ clone 6D11 (Cat. SIG-399810, BioLegend, 1:500); Mouse anti-MBP (Cat. NE1018, Millipore, 1:1,000). Following secondary antibodies were used: Goat anti-mouse IgG (H + L) Alexa Fluor 546 conjugate (Cat. A-11030, Life Technologies, 1:500); Goat anti-mouse IgG (H + L) Alexa Fluor 647 conjugate (Cat. A-21235, Life Technologies, 1:500); Goat anti-rabbit IgG (H + L) Alexa Fluor 488 conjugate (Cat. A-11008, Life Technologies, 1:500); Goat anti-rabbit IgG (H + L) Alexa Fluor 555 conjugate (Cat. A-21428, Life Technologies, 1:500).

### Quantification of the paranodal and nodal length (width) and axon diameter by Caspr and pan Na_v_ immunostaining

Fluorescent images captured of paranodal/nodal domains from frozen sections were measured manually using Imaris software (Bitplane) based on the distance of Caspr and pan Na_v_ (pNa_v_) labelling throughout the dorso-lateral white matter tracts of either thoracic-cervical or lumbo-sacral spinal cords. At least 300 Caspr-positive paranodes and pNa_v_-positive nodes were measured from each of the *ngr1* ^+/+^ and *ngr1* ^−/−^ mice.

### Fluorescence intensity measurement along axo-glial junctions

Fluorescence intensity distribution of Nogo-A and Caspr along axo-glial junction (paranodes) were measured manually using Imaris software (Bitplane) from fluorescence images of paranodal/nodal domains from frozen sections of lumbo-sacral spinal cords of the *ngr1* ^+/+^ and *ngr1* ^−/−^ mice. At least 300 Caspr- and Nogo-A-positive axo-glia junctions were measured from each genotype.

### Myelin preparation

Myelin from naïve and EAE (peak) lumbosacral spinal cords of *ngr1* ^+/+^ and naïve *ngr1* ^−/−^ mice were purified according to published protocol^61, 62^. Briefly, tissues homogenised in 0.32 M sucrose were layered over 0.85 M sucrose (sucrose gradient). These were then ultracentrifuged overnight at 4 °C at 100,000 × g. The myelin interphase was collected with pasteur pipette and washed with ice-cold 20 mM Tris-HCl. Diluted myelin was ultracentrifuged for 1 hour at 100,000 × g to pellet. Crude pellets were then re-suspended in ice-cold 20 mM Tris-HCl and incubated on ice for 10 minutes. Tissues were then re-homogenised and centrifuged for 20 minutes at 12,000 × g (osmotic shock). Pellets were collected and subjected to another two rounds of sucrose gradients and osmotic shocks. Purified myelin was collected and their protein concentrations were measured by BCA colorimetric assay (Thermo-Fischer Scientific).

### Western blot

Protein lysates were prepared from the lumbosacral spinal cords of *ngr1* ^+/+^ and *ngr1* ^−/−^ mice by snap-freezing tissues in liquid nitrogen. Tissue samples were then homogenised in 1X RIPA buffer (Cell Signaling Technologies) with protease (Calbiochem) and phosphatase inhibitors (Calbiochem). The resulting homogenate was then centrifuged at 13,000 rpm for 20 minutes, then the supernatant was collected and protein concentrations were determined using the by BCA colorimetric assay (Thermo-Fischer Scientific).

Either 10 μg (for silver staining), or 5 μg (for immunoblot) of purified myelin or total LSSC lysates were loaded and run on 4–12% Bis-Tris gel (Invitrogen) or 8–20% Tris-Acetate gels (Invitrogen; for Reelin). Silver staining was performed according to manufacturer’s protocol (Bio-Rad). For immunoblot analysis, protein-loaded gels were electrophoretically transferred onto PVDF membranes (Millipore). The membranes were blocked in 5% skim milk in Tris-based saline with 0.1% Tween-20 (TBST) and the primary antibodies diluted in blocking buffer were added and incubated overnight at 4 °C. After thoroughly washing the membranes with TBST, secondary antibodies were added and incubated for 2 hours at room temperature. Immunoreactive proteins were detected using the ECL Prime chemiluminescence kit (GE healthcare). Following primary antibodies were used: Rabbit anti-Caspr (Cat. ab34151, Abcam, 1:20,000); Rabbit anti-Neurofascin 155 (Cat. 15035, Cell Signaling Technology, 1:5,000); Mouse anti-MBP (Cat. ab62631, Abcam, 1:5,000); Mouse anti-CNPase (Cat. MAB3236R, Millipore, 1:5,000); Sheep anti-Nogo-A (Cat. AF3515, R&D systems, 1:2,000); Mouse anti-MOG (Cat. 2116701, Millipore, 1:20,000); Mouse anti-MAG (Cat. MAB1567, Millipore, 1:5,000) Mouse anti-PrP^C^ clone 6D11 (Cat. SIG-399810, BioLegend, 1:1.000); Mouse anti-Reelin (Cat. MAB5366, Millipore, 1:2,000); Mouse anti-βAPP clone 3E9 (Cat. MA1–25489, Thermo-Scientific, 1:1,000); Mouse anti-Actin clone C4 (Cat. MAB1501, Millipore, 1:40,000). Following secondary antibodies were used: HRP conjugated Goat anti-mouse IgG (H + L) (Cat. AB308P, Millipore, 1:20,000); HRP conjugated Goat anti-rabbit IgG (H + L) (Cat. AB307P, Millipore, 1:20,000); HRP conjugated Donkey anti-sheep IgG (H + L) (Cat. HAF016, R&D systems, 1:20,000). The optical density was measured by ImageJ and normalised against anti-Actin immunoreactive loading control bands.

### Electrophysiology

Animals were deeply anaesthetised by sodium pentobarbital (60 mg/kg, i.p.) and underwent trans-cardiac infusion with cold and 95% O_2_/5% CO_2_ saturated high sucrose-modified artificial cerebrospinal fluid (ACSF) containing: sucrose 210 mM; NaHCO_3_ 26 mM; KCl 2.5 mM; CaCl_2_ 1 mM; MgCl_2_ 4 mM; NaH_2_PO_4_ 1.25 mM; D-glucose 10 mM.

#### Spinal cord recordings

The spine from upper cervical to sacral levels was rapidly cut out and placed in ice-cold (4 °C) oxygenated (95% O_2_ + 5% CO_2_) high sucrose-modified ACSF. A laminectomy was performed to expose the whole length of spinal cord. The spinal cord was dissected and the spinal roots and all meninges were removed using microscissors. The spinal cord was then incubated in ACSF containing: NaCl 125 mM; NaHCO_3_ 26 mM; KCl 2.5 mM; CaCl_2_ 2 mM; MgSO_4_ 1.3 mM; NaH_2_PO_4_ 1.25 mM; D-glucose 10 mM at room temperature with 95% O_2_ + 5% CO_2_ for at least 1.5 hours before recording. The upper lumbar segment of spinal cord was placed in the central recording compartment of the recording apparatus, and CAPs were recorded using a modified single sucrose gap chamber as previously described^34^. All solutions used for perfusion were oxygenated by 95% O_2_ + 5% CO_2_ and preheated to 30~32 °C. Electrical pulse stimuli were applied through a PSIU6 photoelectric stimulus isolation unit connected to an S88 stimulator (Grass Instrument Co., USA). The recording electrodes were connected to the headstage of Axoprobe 1 A amplifier (Axon Instruments). The signals were amplified 100 × in DC mode (10 × by Axoprobe 1 A and then 10 × by a custom-made DC preamplifier), processed using Digidata 1322 A, stored on PC and analysed using pClamp software (versions 8.0 and 10, Molecular Devices, USA). The active conduction distance (length of the central compartment of the recording apparatus perfused with oxygenated ACSF) was 7 mm in all experiments and the temperature was 30–32 °C.

#### Optic nerve recordings

the optic nerves were transected behind the eyes within the orbits and the brain with optic nerves attached was quickly removed to ice-cold oxygenated high sucrose-modified ACSF. The optic nerves were removed by transection at the optic chiasm and incubated in ACSF at room temperature for at least 1 hour. The optic nerves were transferred to the recording chamber that was constantly perfused with oxygenated standard ACSF at 36 ± 1 °C. Suction electrodes, attached to the ends of optic nerve for stimulation and recordings, similar to earlier publications by others^35, 43, 63^, were utilised. The distance between the “mouths” of stimulating and recording suction electrodes was measured from photographs taken through the stereomicroscope and used for calculation of conduction velocities of CAP peaks using their latencies. Stimulus-response relationships were taken using 0.05 ms rectangular current pulses ranging from 0 mA to 150 mA with 10 mA increments, and from 100 mA to 1500 mA with 100 mA increments. The 0–150 mA range was often sufficient for achieving a maximal amplitude of the two first peaks of CAPs, while the third, slower conducting peak often required the 100 mA to 1500 mA range. Stimuli were applied via the PSIU6 stimulus isolation units of Grass S88X dual-channel stimulator (Grass Technologies). The recorded signals were amplified and processed using MultiClamp 700B amplifier, DigiData1440 interface and pClamp10 software (all from Molecular devices, USA). The recorded signals were stored on PC computer and analysed offline using pClamp10 software.

### Statistics

Data were analysed using Graph Pad Prism v6.0e. Data represented as mean ± SEM. A two-tailed Student’s t-test was used followed by a *Bonferroni post-hoc* analysis to determine statistical significance unless otherwise specified.

## Electronic supplementary material

Supplementary Information

## References

[1] Caldwell JH, Schaller KL, Lasher RS, Peles E, Levinson SR (2000). Sodium channel Na(v)1.6 is localized at nodes of ranvier, dendrites, and synapses. Proceedings of the National Academy of Sciences of the United States of America.

[2] Peles E, Salzer JL (2000). Molecular domains of myelinated axons. Curr Opin Neurobiol.

[3] Herbert AL, Monk KR (2017). Advances in myelinating glial cell development. Curr Opin Neurobiol.

[4] Bhat MA (2001). Axon-glia interactions and the domain organization of myelinated axons requires neurexin IV/Caspr/Paranodin. Neuron.

[5] Fournier AE, GrandPre T, Strittmatter SM (2001). Identification of a receptor mediating Nogo-66 inhibition of axonal regeneration. Nature.

[6] Wang X (2011). Recovery from chronic spinal cord contusion after Nogo receptor intervention. Ann Neurol.

[7] Park JH (2006). Alzheimer precursor protein interaction with the Nogo-66 receptor reduces amyloid-beta plaque deposition. J Neurosci.

[8] Zai L (2011). Inosine augments the effects of a Nogo receptor blocker and of environmental enrichment to restore skilled forelimb use after stroke. J Neurosci.

[9] Petratos S (2012). Limiting multiple sclerosis related axonopathy by blocking Nogo receptor and CRMP-2 phosphorylation. Brain.

[10] Lee, J. Y. & Petratos, S. Multiple Sclerosis: Does Nogo Play a Role? *Neuroscientist*, doi:10.1177/1073858413477207 (2013). 10.1177/1073858413477207 23423307

[11] Park JH, Strittmatter SM (2007). Nogo receptor interacts with brain APP and Abeta to reduce pathologic changes in Alzheimer’s transgenic mice. Curr Alzheimer Res.

[12] Lee JY, Petratos S (2013). Multiple sclerosis: does Nogo play a role?. Neuroscientist.

[13] Ineichen BV (2017). Nogo-A Antibodies for Progressive Multiple Sclerosis. CNS Drugs.

[14] Lee JY, Biemond M, Petratos S (2015). Axonal degeneration in multiple sclerosis: defining therapeutic targets by identifying the causes of pathology. Neurodegener Dis Manag.

[15] Akbik FV, Bhagat SM, Patel PR, Cafferty WB, Strittmatter SM (2013). Anatomical plasticity of adult brain is titrated by Nogo Receptor 1. Neuron.

[16] McGee AW, Yang Y, Fischer QS, Daw NW, Strittmatter SM (2005). Experience-driven plasticity of visual cortex limited by myelin and Nogo receptor. Science.

[17] Lee H (2008). Synaptic function for the Nogo-66 receptor NgR1: regulation of dendritic spine morphology and activity-dependent synaptic strength. The Journal of neuroscience: the official journal of the Society for Neuroscience.

[18] Karlsson TE (2016). NgR1: A Tunable Sensor Regulating Memory Formation, Synaptic, and Dendritic Plasticity. Cerebral cortex.

[19] Sozmen EG (2016). Nogo receptor blockade overcomes remyelination failure after white matter stroke and stimulates functional recovery in aged mice. Proceedings of the National Academy of Sciences of the United States of America.

[20] Kellner Y (2016). Nogo-A controls structural plasticity at dendritic spines by rapidly modulating actin dynamics. Hippocampus.

[21] Bhagat SM, Butler SS, Taylor JR, McEwen BS, Strittmatter SM (2016). Erasure of fear memories is prevented by Nogo Receptor 1 in adulthood. Mol Psychiatry.

[22] Perrot R, Lonchampt P, Peterson AC, Eyer J (2007). Axonal neurofilaments control multiple fiber properties but do not influence structure or spacing of nodes of Ranvier. J Neurosci.

[23] Hsieh ST (1994). Regional modulation of neurofilament organization by myelination in normal axons. J Neurosci.

[24] Kirkpatrick LL, Brady ST (1994). Modulation of the axonal microtubule cytoskeleton by myelinating Schwann cells. J Neurosci.

[25] Gillespie MJ, Stein RB (1983). The relationship between axon diameter, myelin thickness and conduction velocity during atrophy of mammalian peripheral nerves. Brain Res.

[26] Rios JC (2003). Paranodal interactions regulate expression of sodium channel subtypes and provide a diffusion barrier for the node of Ranvier. J Neurosci.

[27] Peles E (1997). Identification of a novel contactin-associated transmembrane receptor with multiple domains implicated in protein-protein interactions. The EMBO journal.

[28] Hur JY (2012). Identification of novel gamma-secretase-associated proteins in detergent-resistant membranes from brain. J Biol Chem.

[29] Devanathan V (2010). Cellular form of prion protein inhibits Reelin-mediated shedding of Caspr from the neuronal cell surface to potentiate Caspr-mediated inhibition of neurite outgrowth. J Neurosci.

[30] Citron M (1996). Evidence that the 42- and 40-amino acid forms of amyloid beta protein are generated from the beta-amyloid precursor protein by different protease activities. Proc Natl Acad Sci USA.

[31] Quattrocchi CC (2002). Reelin is a serine protease of the extracellular matrix. J Biol Chem.

[32] Prinz M (2004). Intrinsic resistance of oligodendrocytes to prion infection. J Neurosci.

[33] Radovanovic I (2005). Truncated prion protein and Doppel are myelinotoxic in the absence of oligodendrocytic PrPC. J Neurosci.

[34] Salewski RP, Mitchell RA, Shen C, Fehlings MG (2015). Transplantation of neural stem cells clonally derived from embryonic stem cells promotes recovery after murine spinal cord injury. Stem Cells Dev.

[35] Stys PK, Ransom BR, Waxman SG (1991). Compound action potential of nerve recorded by suction electrode: a theoretical and experimental analysis. Brain Res.

[36] Cummins KL, Dorfman LJ, Perkel DH (1979). Nerve fiber conduction-velocity distributions. II. Estimation based on two compound action potentials. Electroencephalography and clinical neurophysiology.

[37] Waxman SG, Bennett MV (1972). Relative conduction velocities of small myelinated and non-myelinated fibres in the central nervous system. Nature: New biology.

[38] Hildebrand C, Remahl S, Persson H, Bjartmar C (1993). Myelinated nerve fibres in the CNS. Prog Neurobiol.

[39] Chang KJ (2014). Glial ankyrins facilitate paranodal axoglial junction assembly. Nat Neurosci.

[40] Halter JA, Clark JW (1993). The influence of nodal constriction on conduction velocity in myelinated nerve fibers. Neuroreport.

[41] Nashmi R, Jones OT, Fehlings MG (2000). Abnormal axonal physiology is associated with altered expression and distribution of Kv1.1 and Kv1.2 K+ channels after chronic spinal cord injury. Eur J Neurosci.

[42] Devaux J (2003). Kv3.1b is a novel component of CNS nodes. J Neurosci.

[43] Allen L (2006). Fructose supports energy metabolism of some, but not all, axons in adult mouse optic nerve. J Neurophysiol.

[44] Freeman B (1978). Myelin sheath thickness and conduction latency groups in the cat optic nerve. J Comp Neurol.

[45] Baltan S (2010). Metabolic vulnerability disposes retinal ganglion cell axons to dysfunction in a model of glaucomatous degeneration. J Neurosci.

[46] Ong HH, Wehrli FW (2010). Quantifying axon diameter and intra-cellular volume fraction in excised mouse spinal cord with q-space imaging. Neuroimage.

[47] Arancibia-Carcamo IL, Attwell D (2014). The node of Ranvier in CNS pathology. Acta Neuropathol.

[48] Golan N (2013). Genetic deletion of Cadm4 results in myelin abnormalities resembling Charcot-Marie-Tooth neuropathy. J Neurosci.

[49] Nguyen T (2009). Axonal protective effects of the myelin-associated glycoprotein. J Neurosci.

[50] Craner MJ (2004). Molecular changes in neurons in multiple sclerosis: altered axonal expression of Nav1.2 and Nav1.6 sodium channels and Na+/Ca2+ exchanger. Proc Natl Acad Sci USA.

[51] Wang H, Allen ML, Grigg JJ, Noebels JL, Tempel BL (1995). Hypomyelination alters K+ channel expression in mouse mutants shiverer and Trembler. Neuron.

[52] Boyle ME (2001). Contactin orchestrates assembly of the septate-like junctions at the paranode in myelinated peripheral nerve. Neuron.

[53] Rosenbluth J (1980). Central myelin in the mouse mutant shiverer. J Comp Neurol.

[54] Mierzwa AJ, Arevalo JC, Schiff R, Chao MV, Rosenbluth J (2010). Role of transverse bands in maintaining paranodal structure and axolemmal domain organization in myelinated nerve fibers: effect on longevity in dysmyelinated mutant mice. J Comp Neurol.

[55] Sinha K, Karimi-Abdolrezaee S, Velumian AA, Fehlings MG (2006). Functional changes in genetically dysmyelinated spinal cord axons of shiverer mice: role of juxtaparanodal Kv1 family K+ channels. J Neurophysiol.

[56] Laquerriere A (2014). Mutations in CNTNAP1 and ADCY6 are responsible for severe arthrogryposis multiplex congenita with axoglial defects. Hum Mol Genet.

[57] Cafferty WB, Strittmatter SM (2006). The Nogo-Nogo receptor pathway limits a spectrum of adult CNS axonal growth. J Neurosci.

[58] Gourdain P, Ballerini C, Nicot AB, Carnaud C (2012). Exacerbation of experimental autoimmune encephalomyelitis in prion protein (PrPc)-null mice: evidence for a critical role of the central nervous system. J Neuroinflammation.

[59] Kim JE, Liu BP, Park JH, Strittmatter SM (2004). Nogo-66 receptor prevents raphespinal and rubrospinal axon regeneration and limits functional recovery from spinal cord injury. Neuron.

[60] Azari MF (2006). Leukemia inhibitory factor arrests oligodendrocyte death and demyelination in spinal cord injury. J Neuropathol Exp Neurol.

[61] Norton WT, Poduslo SE (1973). Myelination in rat brain: method of myelin isolation. J Neurochem.

[62] Larocca, J. N. & Norton, W. T. Isolation of myelin. *Current protocols in cell biology/editorial board, Juan S. Bonifacino… [et al.]* Chapter 3, Unit3 25, doi:10.1002/0471143030.cb0325s33 (2007). 10.1002/0471143030.cb0325s33 18228513

[63] Baltan S (2008). White matter vulnerability to ischemic injury increases with age because of enhanced excitotoxicity. J Neurosci.

