# Supplementary material for: Nogo receptor 1 regulates Caspr distribution at axo-glial units in the central nervous system
Source: Sci Rep. 2017 Aug 21;7:8958. doi: 10.1038/s41598-017-09405-9 (PMC5567129; doi:10.1038/s41598-017-09405-9)
Supplement: Supplementary file 1 — Supplementary Information [file 41598_2017_9405_MOESM1_ESM.pdf]

Supplementary Information

Nogo receptor 1 regulates Caspr distribution at axo-glial units in the central nervous system

Author: Jae Young Lee[1], Min Joung Kim[1], Lijun Li[2a], Alexander A. Velumian[2a, 2c, 2d], Pei Mun Aui[1], Michael G Fehlings[2a, 2b, 2c], Steven Petratos[1]

Authors' addresses:

[1]Department of Medicine, Central Clinical School, Monash University, Prahran, Victoria 3004, Australia;

[2]Krembil Research Institute; [2b]Krembil Neuroscience Centre, University Health Network; [2c]Department of Surgery and [2d]Department of Physiology, Faculty of Medicine, University of Toronto, Toronto, Ontario, Canada;

*Correspondence to: Dr. Steven Petratos

E-mail: steven.petratos@monash.edu

Tel: +613 9902 0191

Supplementary Figures

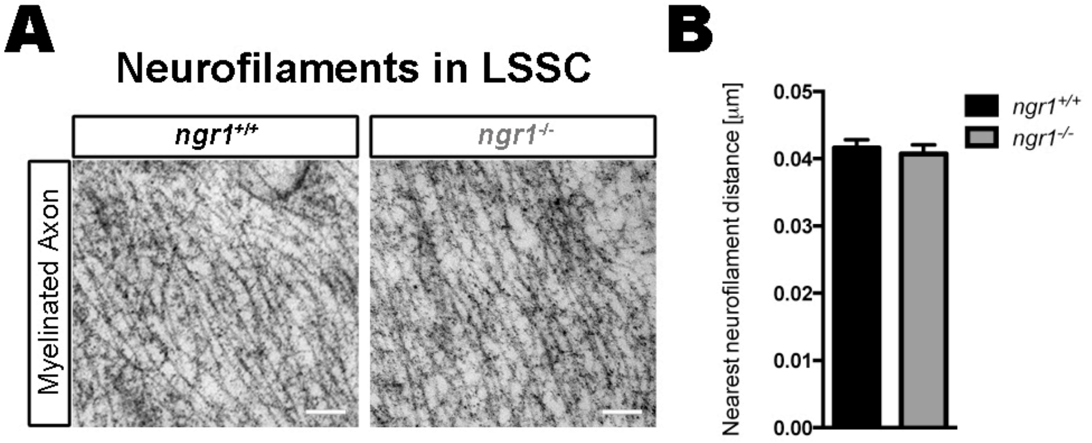

Figure S1. Neurofilament spacing

(A) Ultra-thin (100 nm) electron micrograph longitudinal sections of LSSC from adult *ngr1*$^{+/+}$ and *ngr1*$^{-/-}$ mice showing normal ultra-structure of neurofilaments in both genotypes (Scale bar = 100 nm). (B) Quantification of nearest neighbouring distances between neurofilaments in myelinated axons of descending fibre tracts in *ngr1*$^{+/+}$ and *ngr1*$^{-/-}$ LSSC (*n*=8 for both genotypes).

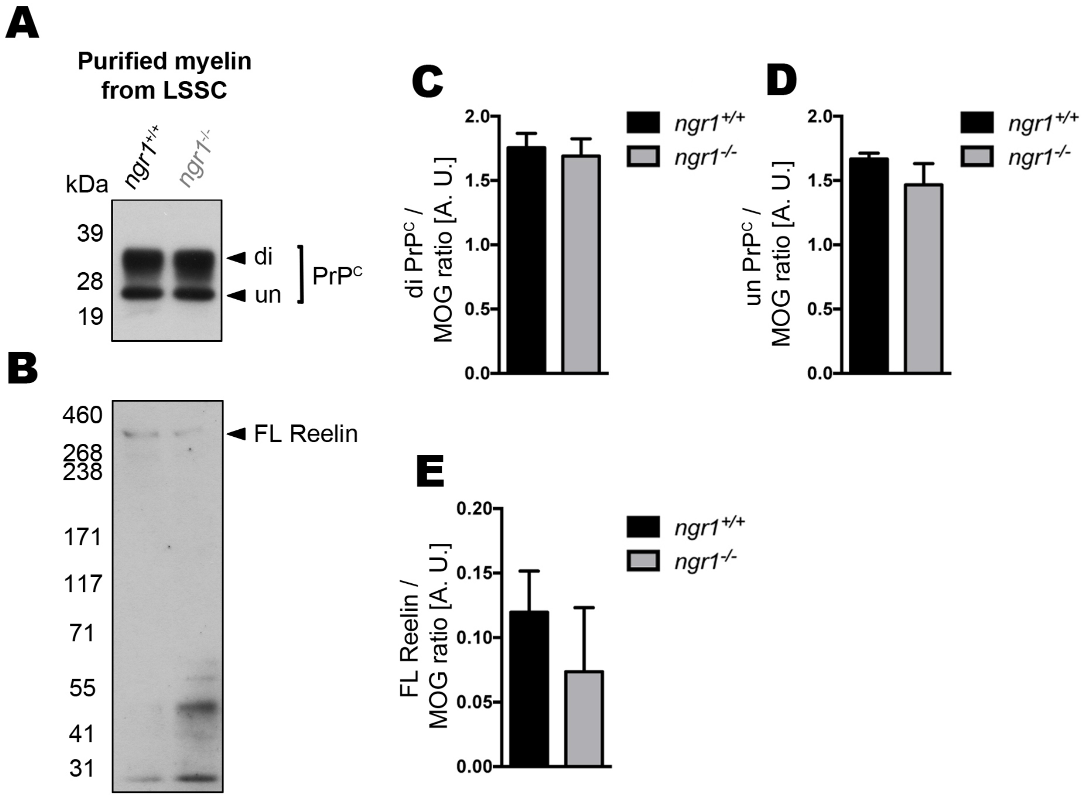

Figure S2. No differences in PrP^C and Reelin levels from isolated *ngr1*$^{+/+}$ and *ngr1*$^{-/-}$ CNS myelin.

(A) Representative immunoblot using anti-PrP^C and (B) anti-Reelin were shown. (C-E) Densitometric quantification (AU) of (C) di PrP^C and (D) un PrP^C; and (E) full-length Reelin (FL Reelin) normalised by the expression level of MOG. (*n*=3 for both genotypes).

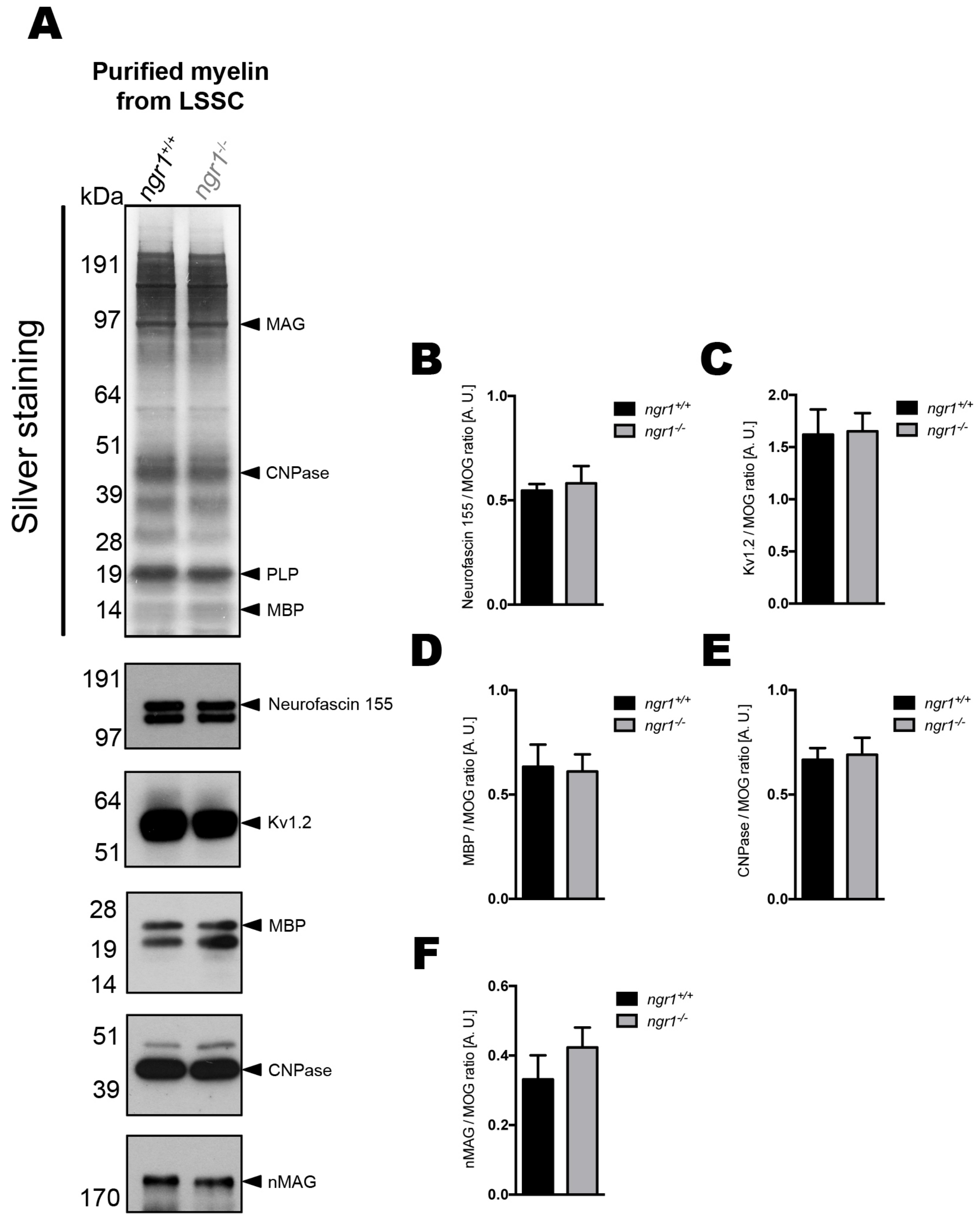

Figure S3. CNS myelin protein levels are not dysregulated in *ngr1*$^{+/+}$ and *ngr1*$^{-/-}$ mice

(A) Purified myelin from LSSCs of *ngr1*$^{+/+}$ and *ngr1*$^{-/-}$ were differentiated one-dimensionally on a 4-12% Bis-Tris gel using a MOPs buffer system. Proteins were visualised by silver staining. Bands consisted of major myelin proteins, which are annotated. Representative immunoblot using anti-Neurofascin 155, anti-K_v1.2 anti-

MBP, anti- CNPase, and anti-nMAG were shown. (B-F) Densitometric quantification (AU) of (B) Neurofascin 155; (C) $K_v1.2$; (D) MBP; (E) CNPase; (F) nMAG normalised by the expression level of MOG (*n*=3 for both genotypes).

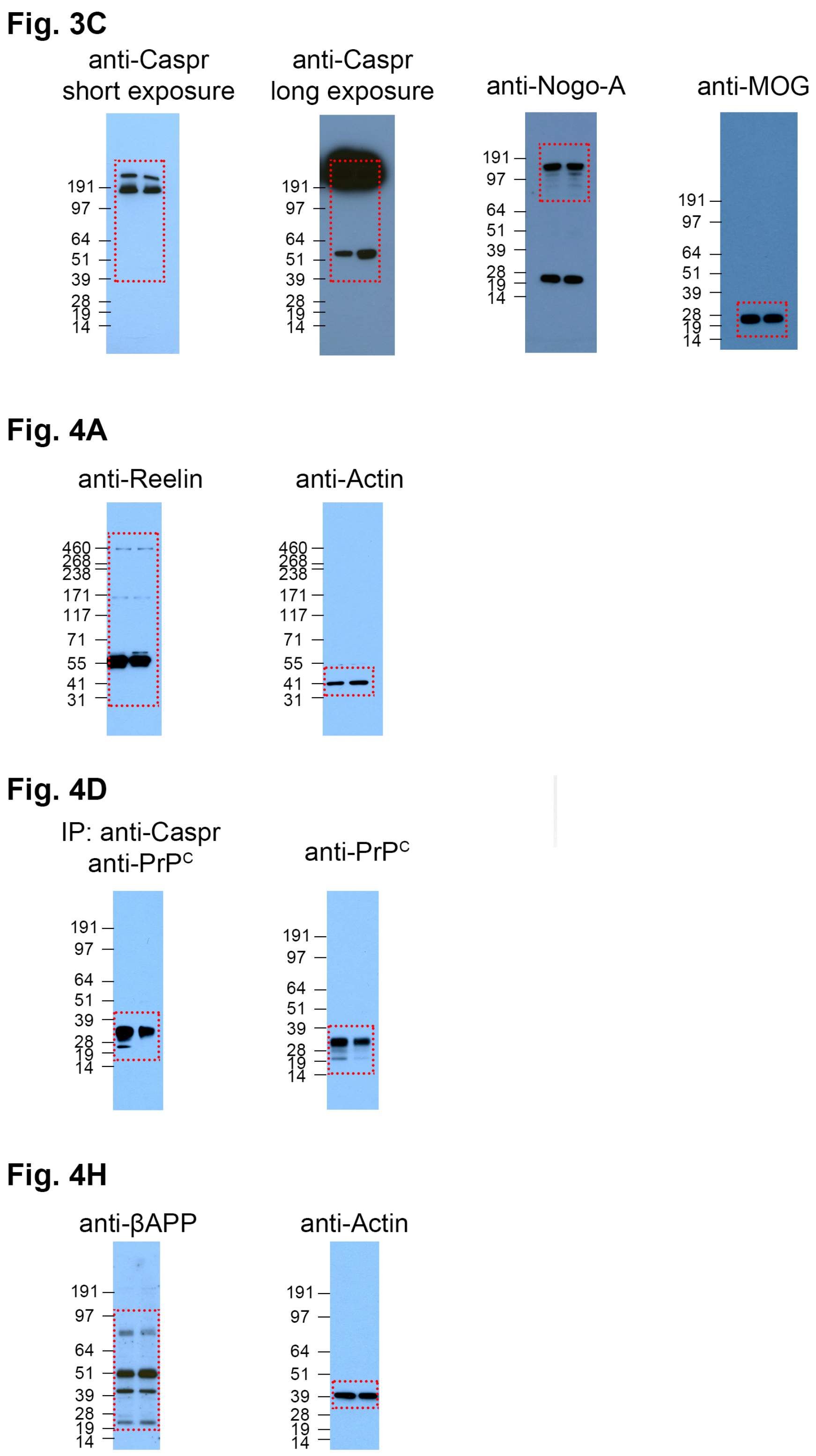

Figure S4. The full immunoblots of Figs 3C, 4A, 4D and 4H

The full immunoblot membranes for the main figures 3C, 4A, 4D and 4H. Dotted lines (red) indicate regions of the blots used in each figure.

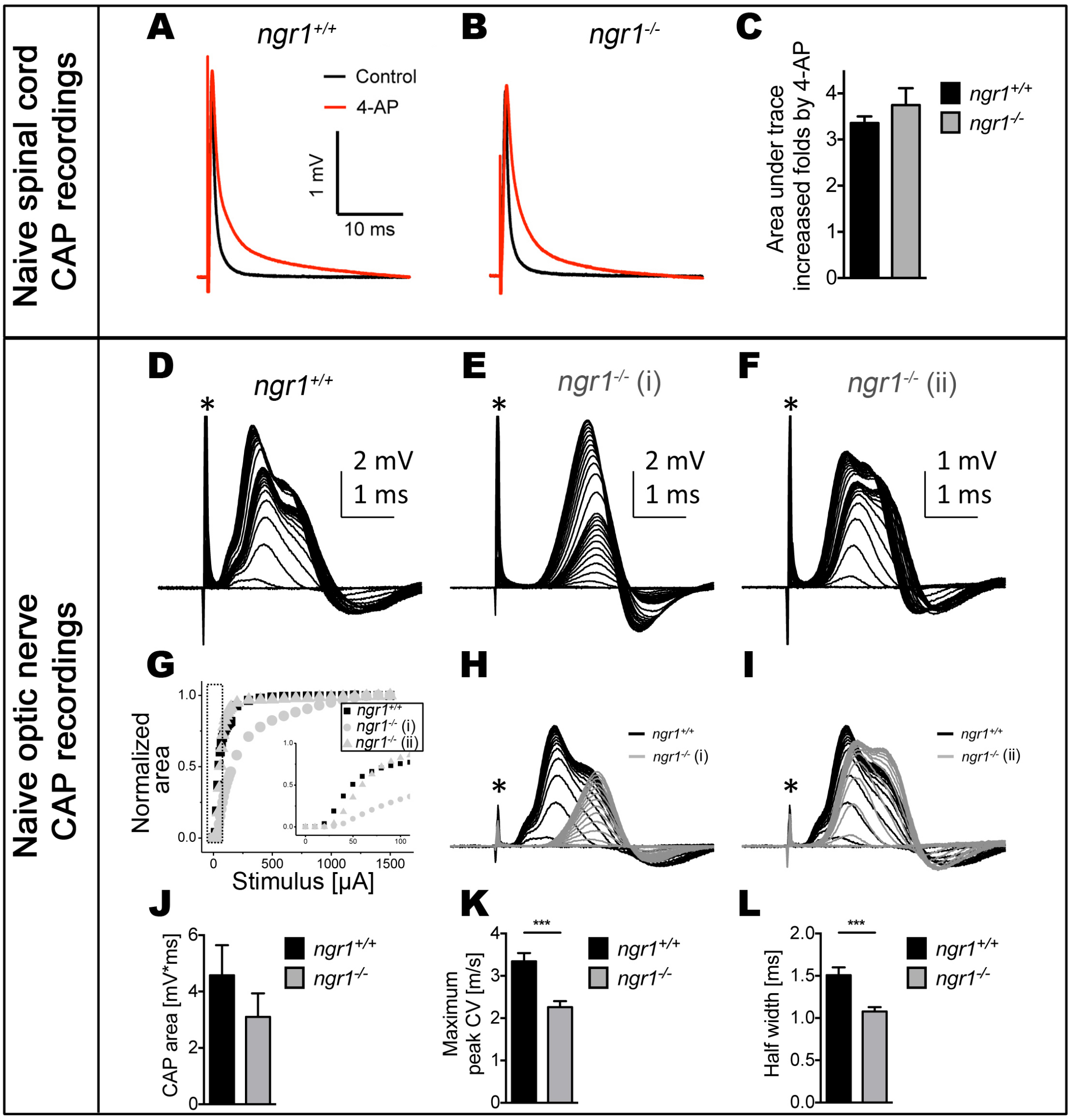

Figure S5. Comparison of compound action potentials (CAPs) in *ngr1*$^{+/+}$ and *ngr1*$^{-/-}$ mice

(A-C) No significant differences between naïve *ngr1*$^{+/+}$ and *ngr1*$^{-/-}$ spinal cord white matter in the sensitivity of compound action potentials to K_v blocker, 4-AP. Representative superimposed sucrose gap recordings of CAPs from naïve spinal cords of *ngr1*$^{+/+}$ and *ngr1*$^{-/-}$ mice, with and without 0.5 mM 4-AP treatment (red and black traces, respectively). The CAP area increased following 4-AP administration in *ngr1*$^{+/+}$ and *ngr1*$^{-/-}$ mice (*n*=6 for *ngr1*$^{+/+}$ and *n*=7 for *ngr1*$^{-/-}$). (D-L) Optic nerve CAPs of *ngr1*$^{+/+}$ and *ngr1*$^{-/-}$ mice recorded with suction electrodes. (D-F)

Representative CAPs evoked by varied stimulus intensities in optic nerves. Shown are superimposed traces evoked in the range 0-150 μA at 10 μA step increments and in the 200-1500 μA range at 100 μA step increments. The stimulus artifacts are marked by asterisks (*). (D) *ngr1*$^{+/+}$ CAPs exhibited characteristic 3 peaks which differed significantly in their conduction velocities (peak 1 *vs.* peak 2: p = 5.5918E-8; peak 2 *vs.* peak 3: p = 1.53541E-8; peak 1 *vs.* peak 3: p = 3.25849E-11). (F) In *ngr1*$^{-/-}$ mice, CAPs had 3 identifiable peaks in only 6 out of 14 optic nerves. In these 6 optic nerves, the 3 peaks differed significantly in their CV (peak 1 vs. peak 2: p = 6.16255E-9; peak 2 vs. peak 3: p = 5.73343E-6; peak 1 vs. peak 3: p = 5.33254E-12). (E) The other 8 *ngr1*$^{-/-}$ optic nerve CAPs had a single-peak appearance at all stimulation intensities. (G) Stimulus-response relationships of CAP area for *ngr1*$^{+/+}$, *ngr1*$^{-/-}$ (i) and *ngr1*$^{-/-}$ (ii) CAPs shown in D-F. (H, I) Superimposed CAPs evoked in the 0-150 mA stimulus intensity range from *ngr1*$^{+/+}$ (black traces) and *ngr1*$^{-/-}$ optic nerves (gray traces): *ngr1*$^{-/-}$ (i) (H) and *ngr1*$^{-/-}$ (ii) (I), all traces selected from panels D-F respectively, to further illustrate the difference in CAP shapes and peak latencies between *ngr1*$^{+/+}$ and *ngr1*$^{-/-}$ (i) and *ngr1*$^{-/-}$ optic nerves. (J-L) Statistical comparisons of *ngr1*$^{+/+}$ and *ngr1*$^{-/-}$ CAPs, with p values of independent t-tests shown for all pairs. Shown are commonly used overall parameters describing the optic nerve CAPs evoked by maximal stimulation intensities (1500 mA): (J) CAP area, (K) CV of CAP's maximal peak and (L) CAP half-width.
